# Selumetinib, an Oral Anti-Neoplastic Drug, May Attenuate Cardiac Hypertrophy via Targeting the ERK Pathway

**DOI:** 10.1371/journal.pone.0159079

**Published:** 2016-07-20

**Authors:** Chen Li, Zhongxiu Chen, Hao Yang, Fangbo Luo, Lihong Chen, Huawei Cai, Yajiao Li, Guiying You, Dan Long, Shengfu Li, Qiuping Zhang, Li Rao

**Affiliations:** 1 Department of Cardiology, West China Hospital of Sichuan University, Chengdu, Sichuan, China; 2 Department of Rehabilitation, Community Health Center of Shuangnan Wuhou District, Chengdu, Sichuan, China; 3 Department of Biochemistry and Molecular Biology, West China School of Preclinical and Forensic Medicine, Sichuan University, Chengdu, Sichuan, China; 4 Department of Nuclear Medicine, West China Hospital of Sichuan University, Chengdu, Sichuan, China; 5 Key Laboratory of Transplant Engineering and Immunology, West China Hospital of Sichuan University, High-tech Zone, Chengdu, Sichuan, China; 6 King’s College London British Heart Foundation Centre of Research Excellence, Cardiovascular Division, London, United Kingdom; Universidade Federal do Rio de Janeiro, BRAZIL

## Abstract

**Aims:**

Although extracellular-regulated kinases (ERK) are a well-known central mediator in cardiac hypertrophy, no clinically available ERK antagonist has been tested for preventing cardiac hypertrophy. Selumetinib is a novel oral MEK inhibitor that is currently under Phase II and Phase III clinical investigation for advanced solid tumors. In this study, we investigated whether Selumetinib could inhibit the aberrant ERK activation of the heart in response to stress as well as prevent cardiac hypertrophy.

**Methods and Results:**

In an *in vitro* model of PE-induced cardiac hypertrophy, Selumetinib significantly inhibited the ERK activation and prevented enlargement of cardiomyocytes or reactivation of certain fetal genes. In the pathologic cardiac hypertrophy model of ascending aortic constriction, Selumetinib provided significant ERK inhibition in the stressed heart but not in the other organs. This selective ERK inhibition prevented left ventricular (LV) wall thickening, LV mass increase, fetal gene reactivation and cardiac fibrosis. In another distinct physiologic cardiac hypertrophy model of a swimming rat, Selumetinib provided a similar anti-hypertrophy effect, except that no significant fetal gene reactivation or cardiac fibrosis was observed.

**Conclusions:**

Selumetinib, a novel oral anti-cancer drug with good safety records in a number of Phase II clinical trials, can inhibit ERK activity in the heart and prevent cardiac hypertrophy. These promising results indicate that Selumetinib could potentially be used to treat cardiac hypertrophy. However, this hypothesis needs to be validated in human clinical trials.

## Introduction

Cardiac hypertrophy is an increase in the heart size in response to physiological or pathological stress, such as extensive physical exercise, hypertension, valvular disorder or coronary artery disease[1]. Although cardiac hypertrophy was initially regarded as a compensatory response to changes in the mechanical load, accumulating evidence suggests that, in most instances, hypertrophy is a maladaptive process accompanied by fetal gene upregulation, myocardial fibrosis, cardiac dysfunction and, eventually, a higher incidence of clinical events[2,3]. Cardiac hypertrophy is mediated by a variety of intracellular signaling cascades[4,5]. Among these pro-hypertrophic signaling pathways, the extracellular-regulated kinases (ERKs) are a well-known central mediator[6,7]. The ERK pathway is activated in response to every stress- and agonist-induced hypertrophic stimulus examined to date, and blocking the ERK signaling pathway prevents against cardiac hypertrophy in vitro and in vivo[8–11]. Unfortunately, the ERK inhibitors or genetic modification approaches used in these cell and animal studies have been far from providing a clinically available treatment option for cardiac hypertrophy.

Although there is no current clinically feasible anti-hypertrophic drug targeting the ERK pathway, several ERK inhibitors have been in clinical development for cancer[12,13]. Among them, Selumetinib (AZD6244 and ARRY-142886; AstraZeneca, Manchester,UK) is a potent, selective, non-ATP-competitive oral MEK1/2 inhibitor that is currently under Phase II and Phase III clinical investigation[14]. Although it was initially designed as an anti-cancer drug, a recent animal study showed that Selumetinib has cardiac protection effects in a murine model of LMNA cardiomyopathy[15]. However, the effect of Selumetinib in normal and hypertrophic heart has still been unclear. In this study, we aimed to investigate whether Selumetinib could inhibit aberrant ERK activation upon stress and prevent cardiac hypertrophy.

## Materials and Methods

### Cell culture and experimental procedure

Primary cultures of neonatal rat cardiomyocytes (NRCs) were established according to a previously published procedure [16]. After treatment with PD98059 (Cayman Chemicals, USA) or Selumetinib (AZD6244, Cayman Chemicals) at a final concentration of 50 μM (PD98059) or 500 nM (AZD624) for 30 min, the cells were stimulated with phenylephrine (PE, Tocris Bioscience) at a final concentration of 100μM for 24 or 48 h in serum-free media. Experiments had been performed also in tumor and non-tumor cell-lines. Detailed protocols and methods were described in the online data supplement.

### Rat and treatment protocols

Detailed protocols for establishing ascending aortic constriction (AAC) and swimming hypertrophy models were described in the online data supplement. The animal procedures were approved by the Institutional Animal Care and Use Committee of Sichuan University. Selumetinib (AZD6244; Cayman Chemicals) was stored at a concentration of 20 mg/mL in dimethyl sulfoxide (DMSO; Sigma) and delivered at a dose of 1 mg/kg/day (dissolved in 20% anhydrous ethanol and 80% 5% glucose solution) by intraperitoneal injection using a 25 G 5/8 syringe. In AAC hypertrophy models, treatment group received Selumetinib starting at 1 week post AAC surgery and continuing until 5 weeks post AAC surgery. The control group received the same volume of placebo consisted of DMSO. At the end of 5 weeks' drug treatment, randomly 6 of 10 rats in each group were sacrificed and tissue samples were harvested. The remaining 4 rats were left for long-term follow up using echocardiogram. In swimming hypertrophy models, Selumetinib were administered throughout the 8 weeks' follow up. All rats were sacrificed at the end of the follow up.

### Measurement of Cardiac Hypertrophy Phenotype

To measure the cardiac hypertrophy phenotype in the cells and rat models, we used transthoracic echocardiography, quantitative real-time RT–PCR, Western blot and histopathological analysis. The detailed methods were described in the online data supplement.

### Statistical Analysis

All statistical analyses were performed using SPSS version 19.0 (SPSS, Inc, Chicago, IL). Data were obtained from separate experiments and expressed as the means ± SEM. Data for echocardiography, LVW/BW and LVW/TL from pressure overload-induced cardiac hypertrophy and swimming exercise-induced cardiac hypertrophic model were expressed as the means ± standard deviation (SD). Comparisons among different treatments in the rats and cardiomyocytes were evaluated using one-way ANOVA. Statistical significance was assumed for p < 0.05.

## Results

### Selumetinib inhibits ERK1/2 activation upon stress and cardiac hypertrophy in NRCs

**Fig 1:**
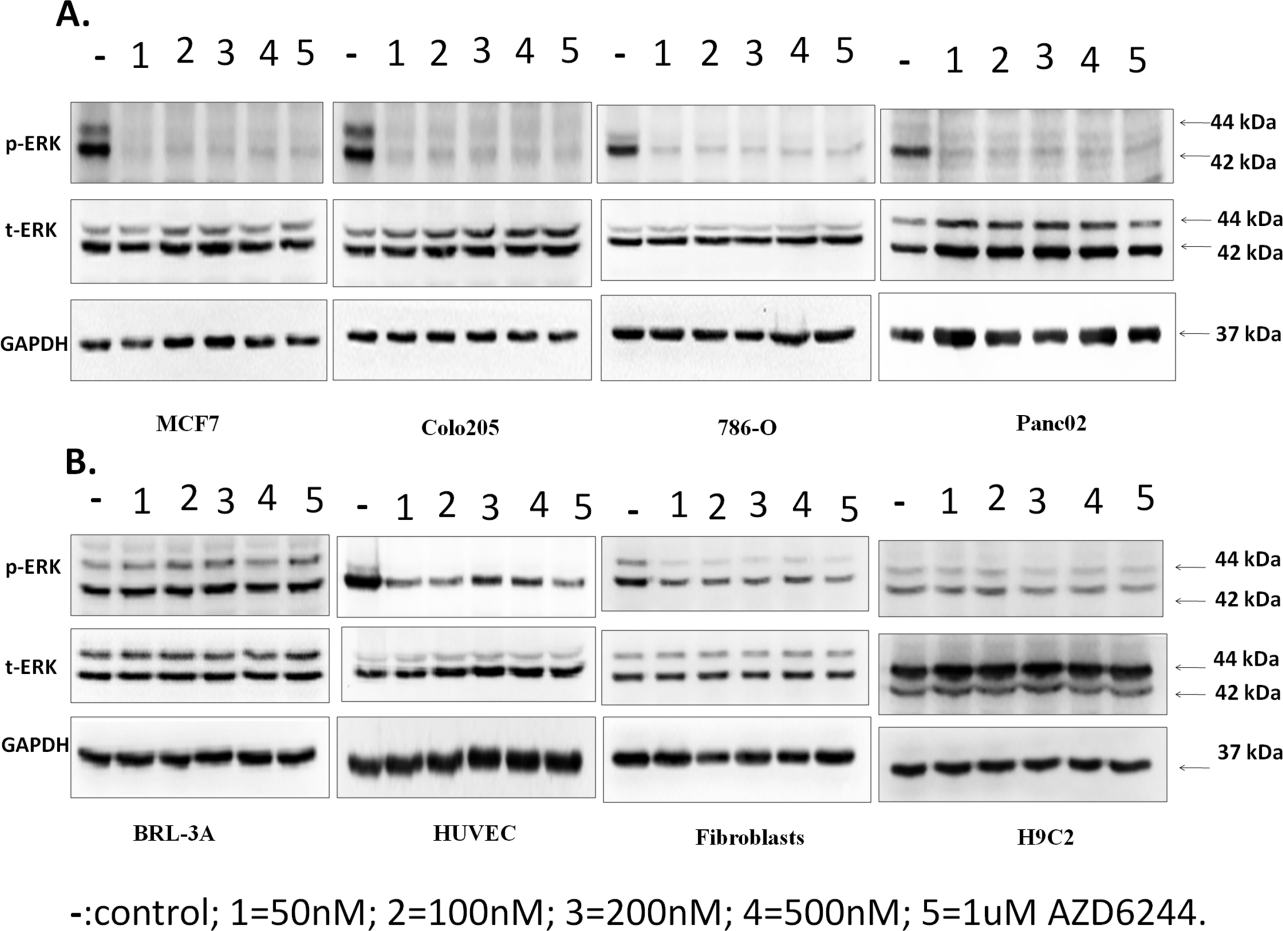
The ERK inhibition effect with AZD6244 was significant in neoplastic cells and mild in non-neoplastic cells. **(A).**Representative immunoblots using antibodies against phosphorylated ERK1/2 (pERK1/2) and total ERK1/2 (ERK1/2) to probe proteins extracted from MCF7, Colo205, 786-O, and Panc02 cell lysates treated with 50 nM, 100 nM, 200 nM, 500 nM, and 1μM AZD or without AZD, n = 3. **(B).**Representative immunoblots using antibodies against phosphorylated ERK1/2 (pERK1/2) and total ERK1/2 (ERK1/2) to probe proteins extracted from BRL-3A, HUVEC, fibroblasts, and H9C2 cell lysates treated with50 nM, 100 nM, 200 nM, 500 nM, and 1μM AZD or without AZD, n = 3.

Previous studies suggested Selumetinib was a potent ERK inhibitor in tumor cell, especially those carrying Ras mutation. However, its effect in normal or stressed cardiomyocytes was not clearly characterized. In our study, we found that a very small dosage of Selumetinib was sufficient to block ERK activity in tumor cells(Fig 1A). However, in non-tumor cell-lines, the ERK inhibition effect of Selumetinib was much milder (Fig 1B).

**Fig 2:**
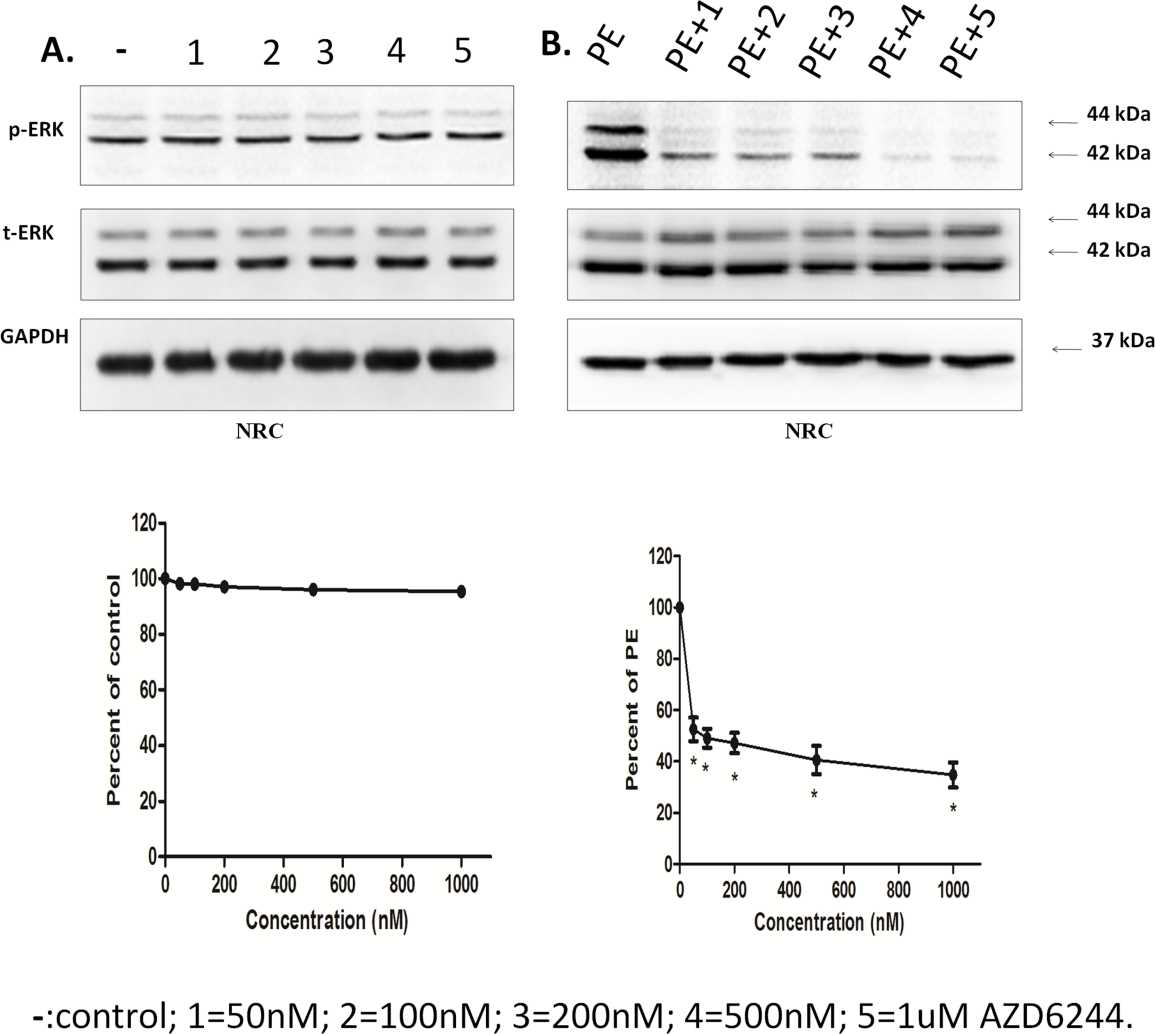
The ERK inhibition effect with AZD6244 was mild in normal cardiomyocytes, but it was more significant in hypertrophic hearts. **(A).**Upper Panel: Representative immunoblots using antibodies against phosphorylated ERK1/2 (pERK1/2) and total ERK1/2 (ERK1/2) to probe proteins extracted from NRCs lysates treated with 50 nM, 100 nM, 200 nM, 500 nM, and 1μM AZD or without AZD. Lower Panel: the percent of pERK/tERK normalized to control, n = 3. **(B).**Upper Panel: representative immunoblots using antibodies against phosphorylated ERK1/2 (pERK1/2) and total ERK1/2 (ERK1/2) to probe proteins extracted from NRCs lysates treated with PE and AZD or without AZD. Lower Panel: the percent of pERK/tERK normalized with PE. * Significantly different from PE(P<0.001, n = 3).

More importantly, we found that Selumetinib had different effects on normal and stressed cardiomyocytes. In normal NRCs, no significant ERK inhibition was observed even though the concentration of Selumetinib was as high as 1000 nM (Fig 2A). But the ERK inhibition effect of Selumetinib was much more extensive in stressed NRCs. A very small dose of Selumetinib (50 nM) was sufficient to induce significant ERK inhibition in NRCs treated with PE (Fig 2B).

**Fig 3:**
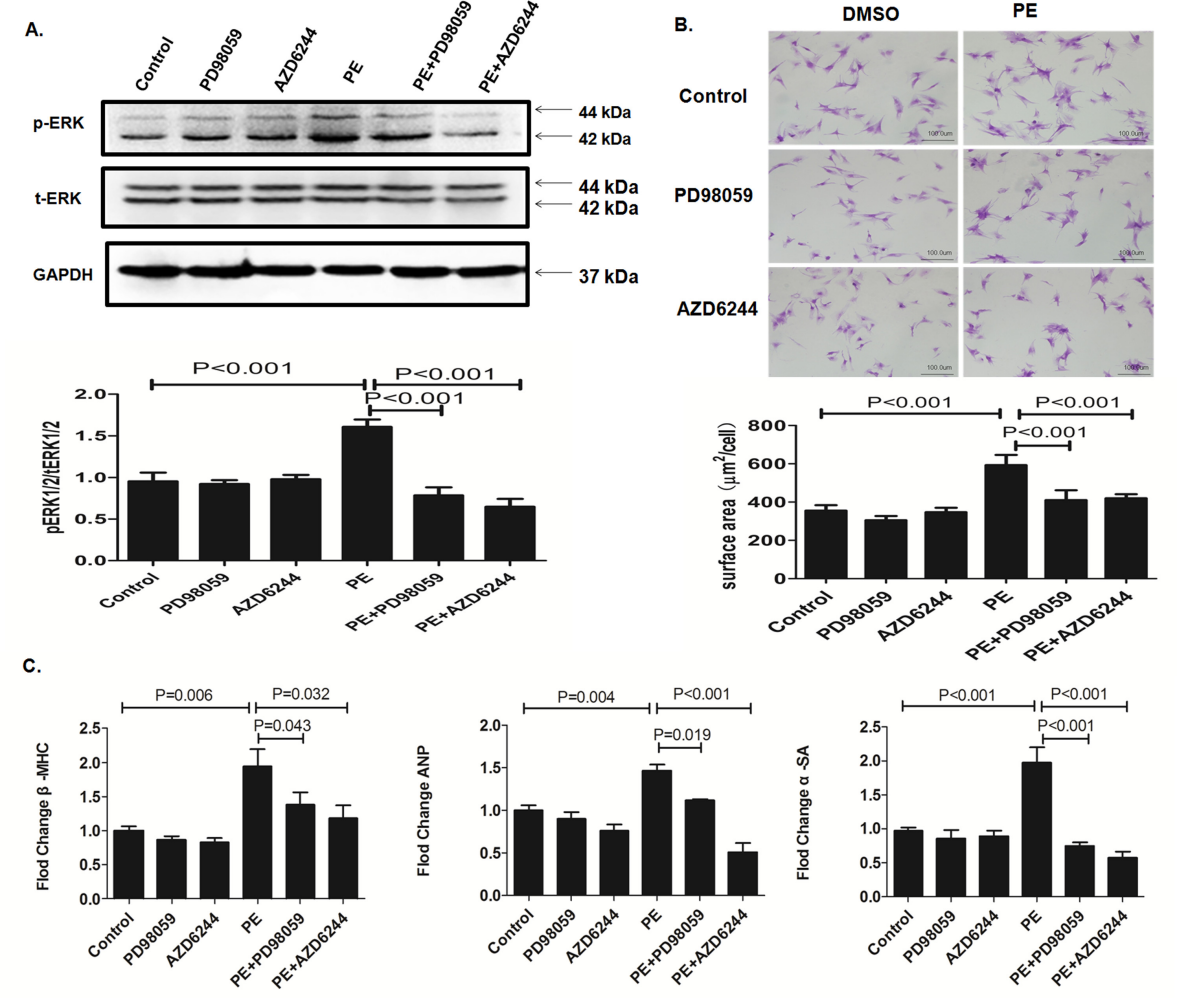
Selumetinib inhibits ERK1/2 activation upon stress and cardiac hypertrophy in NRCs. **(A).** Representative immunoblots using antibodies against phosphorylated ERK1/2 (pERK1/2) and total ERK1/2 (tERK1/2) to probe proteins extracted from NRCs for indicated treatments. The bar graph shows the means ± SEM signals of the pERK/tERK (relative expression from immunoblots of n = 5). **(B).** Representative micrographs of cultured NRCs for indicated treatments. The bar graph shows the means ± SEM of the mean cellular area (n = 3, each time, 90 cells were randomly selected and measured). **(C).** Bar graphs indicate the expression levels of β-MHC, ANP and α-SMA in NRCs for indicated treatments (n = 5).

To investigate the anti-hypertrophic effect of Selumetinib in cardiomyocytes, we used an *in vitro* model of PE-induced cardiac hypertrophy. NRCs treated with PE had significantly higher ERK activity. Both PD98059, a widely used conventional MEK inhibitor, and Selumetinib were able to block the ERK activation caused by PE (Fig 3A). Induction of the hypertrophic responses was monitored by mean cell surface area measurements. NRCs treated with PE demonstrated a larger mean cellular area. Co-treatment with PD98059, or Selumetinib significantly prevented the hypertrophic phenotype (Fig 3B). In addition, increased expressions of cardiac hypertrophy markers, including β- myosin heavy chain(β-MHC), atrial natriuretic peptide (ANP) and α-smooth muscle actin (α-SMA), were observed in NRCs treated with PE. Co-treatment with PD98059 or Selumetinib significantly attenuated the over-expression of these hypertrophy markers. (Fig 3C)

**Fig 4:**
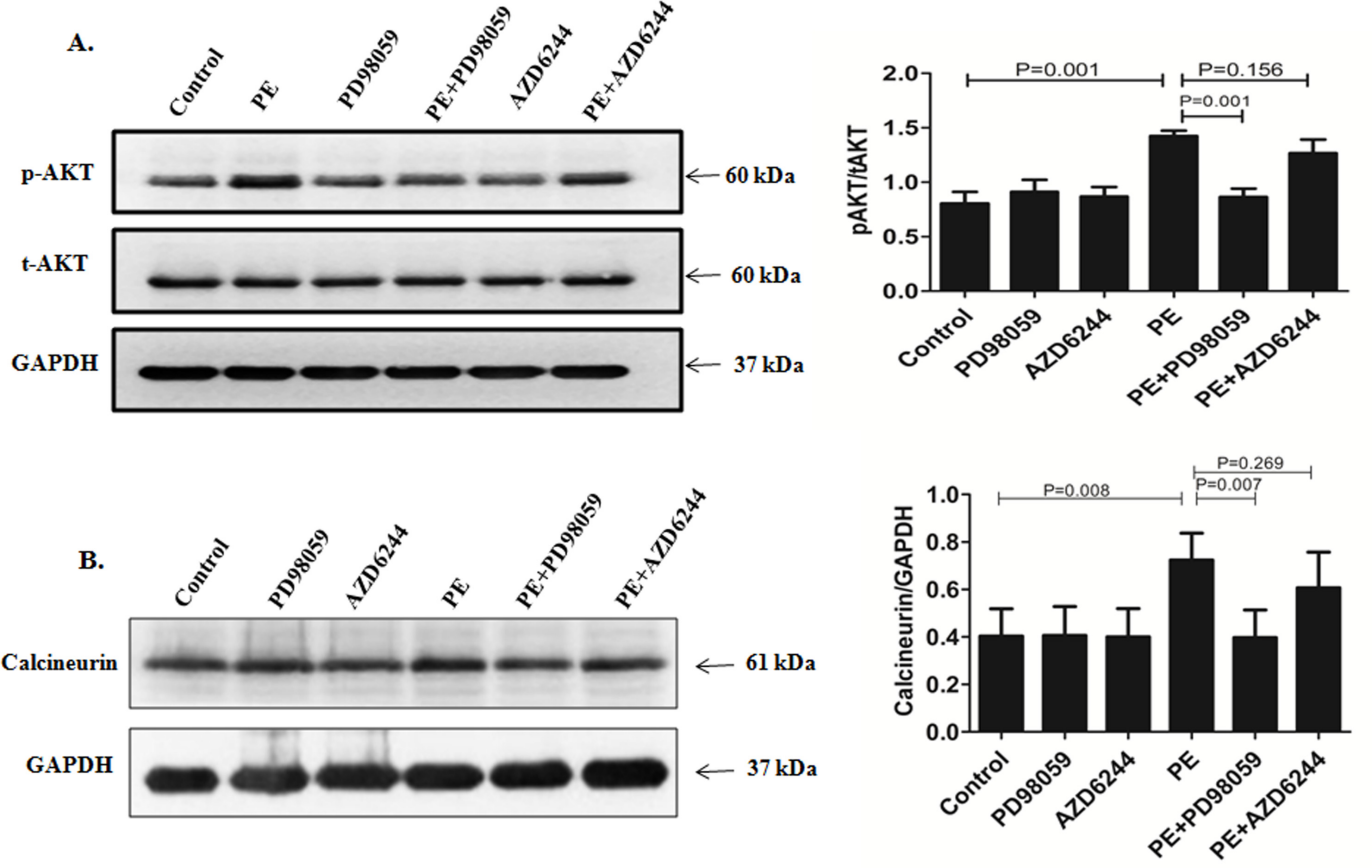
Unlike PD98059, there was no significant AKT activation and calcineurin block for Selumetinib. **(A).** Representative immunoblots using antibodies against phosphorylated AKT (pAKT) and total AKT (tAKT) to probe proteins extracted from NRCs for indicated treatments. The bar graph shows the means ± SEM signals of the pAKT/total AKT (relative expression from immunoblots of n = 3). **(B).** Representative immunoblots using antibodies against calcineurin. The bar graph shows the means ± SEM signals of the calcineurin/GAPDH (relative expression from immunoblots of n = 3).

To further confirm Selumetinib attenuated cardiac hypertrophy via targeting the ERK pathway, other important signaling pathways involved in cardiac hypertrophy were also tested. In our study, Selumetinib selectively inhibit ERK while PD98059 affected not only ERK but also AKT and calcineurin (Fig 4). This was consistent with previous reports that Selumetinib was a highly selective MEK 1/2 inhibitor affecting mainly ERK 1/2[14].

### Selumetinib attenuates cardiac hypertrophy in an ascending aortic constriction rat model

**Fig 5:**
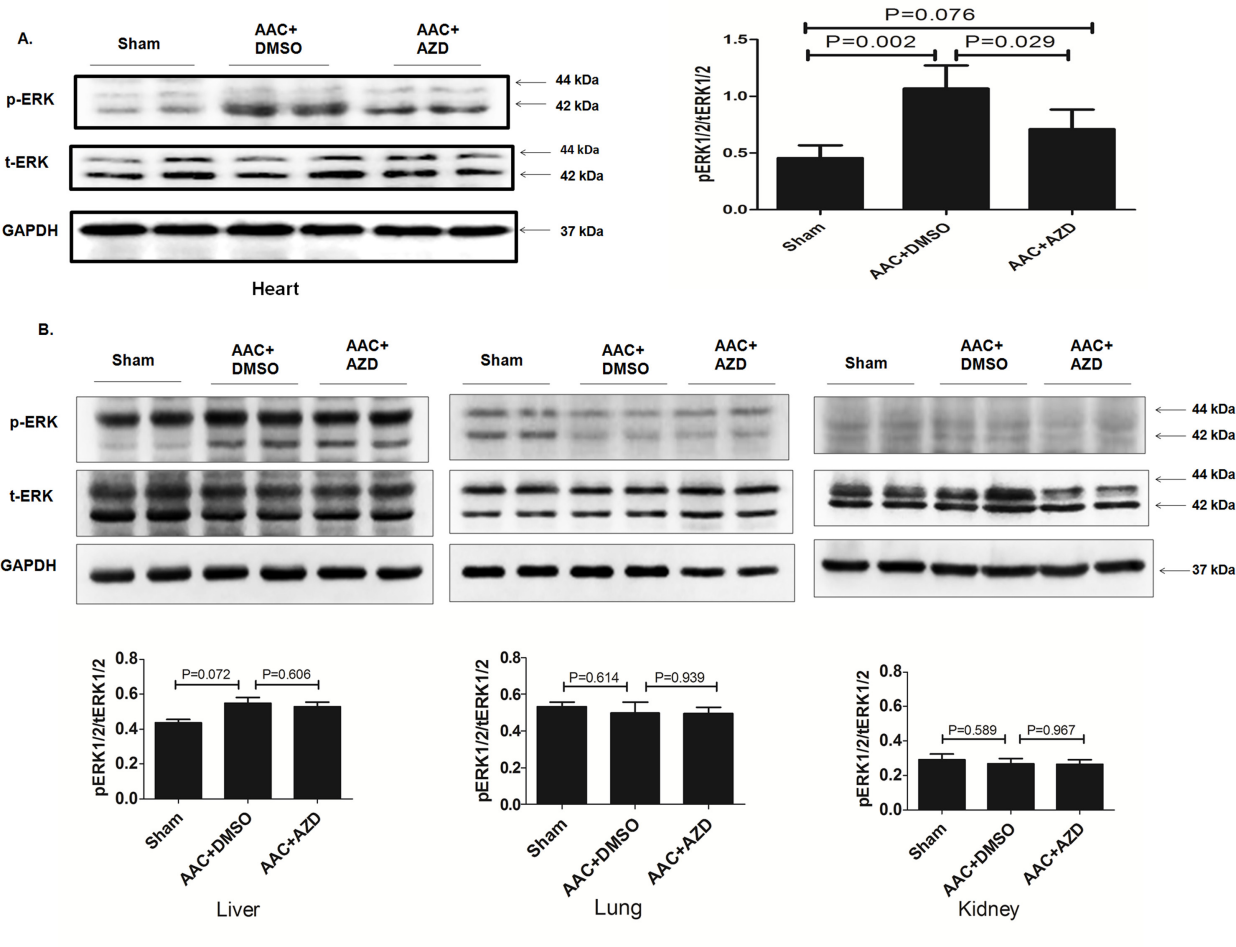
Selumetinib inhibits ERK1/2 activation upon stress in an AAC rat model of cardiac hypertrophy at 5 weeks’ follow-up. **(A).** Representative immunoblots using antibodies against pERK1/2 and tERK1/2 to probe proteins extracted from heart of rats that underwent sham, AAC and AAC plus Selumetinib procedures. The bar graph shows the means ± SEM signals for pERK/tERK (relative expression from immunoblots of n = 5). **(B).** Representative immunoblots using antibodies against pERK1/2 and tERK1/2 to probe proteins extracted from the liver, lung and kidney of rats for indicated procedures. The bar graph shows the means ± SEM signals of pERK/tERK, n = 3, P>0.05.

The *in vitro* cell study suggested that Selumetinib could prevent cardiac hypertrophy caused by chemical stimulation. We also tried to test the anti-hypertrophy effect of Selumetinib in animal models. Ascending aortic constriction was a widely used animal model for pressure overload induced left ventricular (LV) hypertrophy[3]. Rats that underwent AAC had significantly higher ERK activity in the heart but not in the liver, lung or kidney. Selumetinib significantly prevented the aberrant ERK activation in the heart, but it had a minimal effect on the other organs with normal ERK activity (Fig 5).

**Fig 6:**
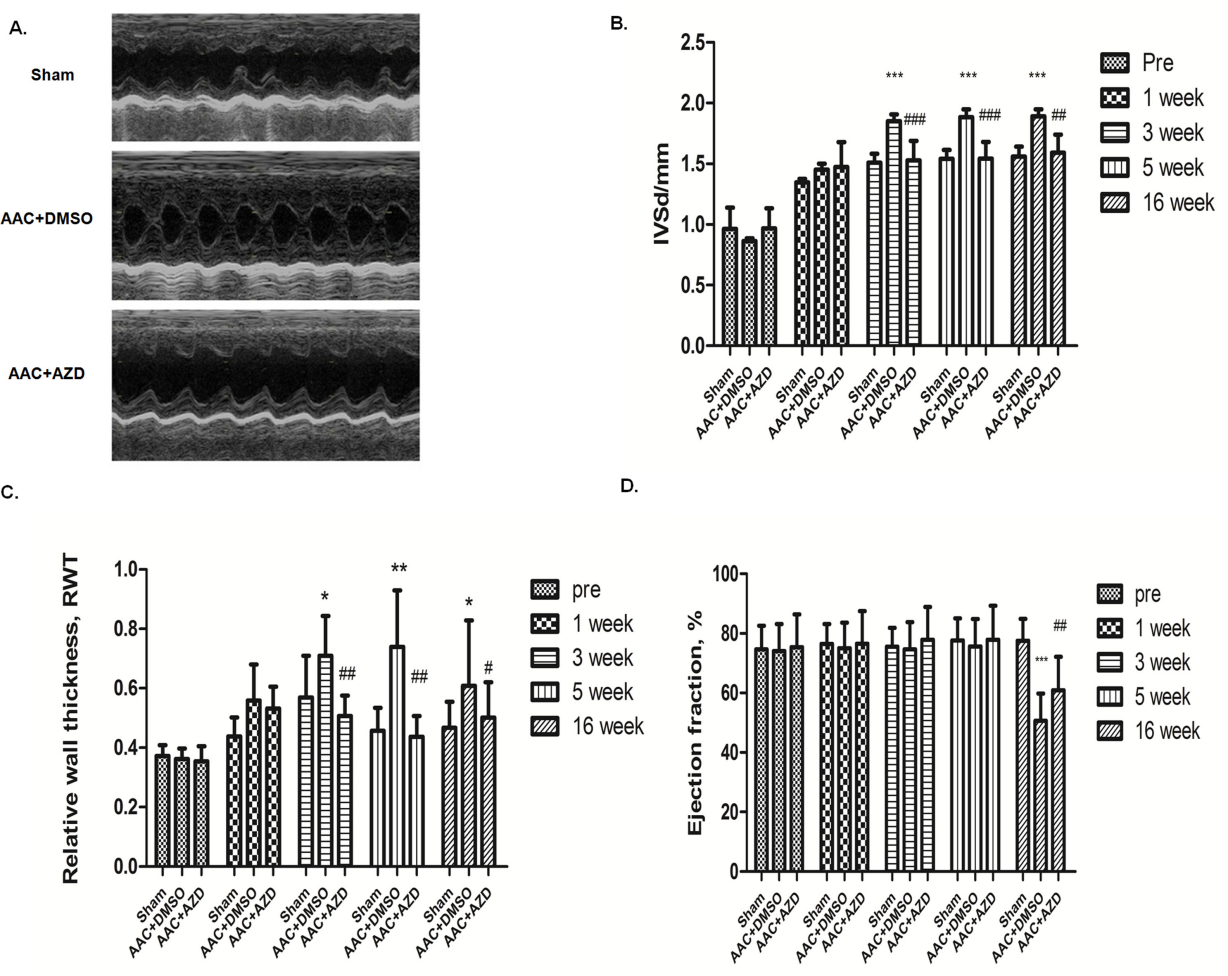
Selumetinib attenuates cardiac hypertrophy induced by AAC assessed by echocardiographic parameters. **(A).** Representative charts of trans-thoracic M-mode echocardiogram of the LV at 5 weeks’ follow-up. **(B).** The diastolic inter-ventricular septal thickness of rats for indicated procedures during follow-up. **(C).** The relative wall thickness of rats for indicated treatments during follow-up. **(D).** The left ventricular ejection fraction of rats for indicated treatments during follow-up. The values are the means ± SD, n = 10 for three groups at Pre, 1, 3, and 5 weeks, and n = 4 for sham and AAC+AZD group, n = 2 for AAC+DMSO group at 16 weeks. *, ** and ***Significantly different from the sham group (P<0.05, P<0.01 and P<0.001 respectively) and #, ## and ### significantly different from the AAC+DMSO group (P<0.05, P<0.01 and P<0.001 respectively).

Rats that underwent AAC demonstrated cardiac hypertrophy, as evidenced by the greater LV mass and thicker LV wall compared with sham rats. By contrast, Selumetinib significantly protected the animals from pressure overload-induced ERK activation, reducing the heart mass and wall thickness (Fig 6, Table 1).

**Table 1:** The changes in the echocardiogram parameters in rats in the AAC group.

Item	Sham	AAC+DMSO	AAC+AZD
HR(bpm)	400 ± 29	414 ± 28	388 ± 88
IVSd(mm)	1.54 ± 0.07	1.89 ± 0.06*	1.54 ± 0.14^#^
LVPWd(mm)	1.50 ± 0.12	1.85 ± 0.11*	1.53 ± 0.21^#^
LVIDd(mm)	5.70 ± 1.29	5.47 ± 0.86	6.70 ± 1.37
LVEDV(ml)	0.48 ± 0.27	0.41 ± 0.18	0.74 ± 0.46
EF(%)	77.61 ± 7.40	75.64 ± 9.16	77.89 ± 11.38

The changes in the echocardiogram parameters in rats in the AAC group at 5 weeks’ follow-up. The values are the mean ± SD, n = 6.

*: p<0.01 compared with sham

#: p<0.01 compared with AAC+DMSO.

HR: heart rate; IVSd: end-diastolic inter-ventricular septal thickness; LVPWd: end-diastolic left ventricular posterior wall thickness; LVIDd: end-diastolic left ventricular internal dimension; LVEDV: left ventricular end-diastolic volume; and EF: ejection fraction.

**Fig 7:**
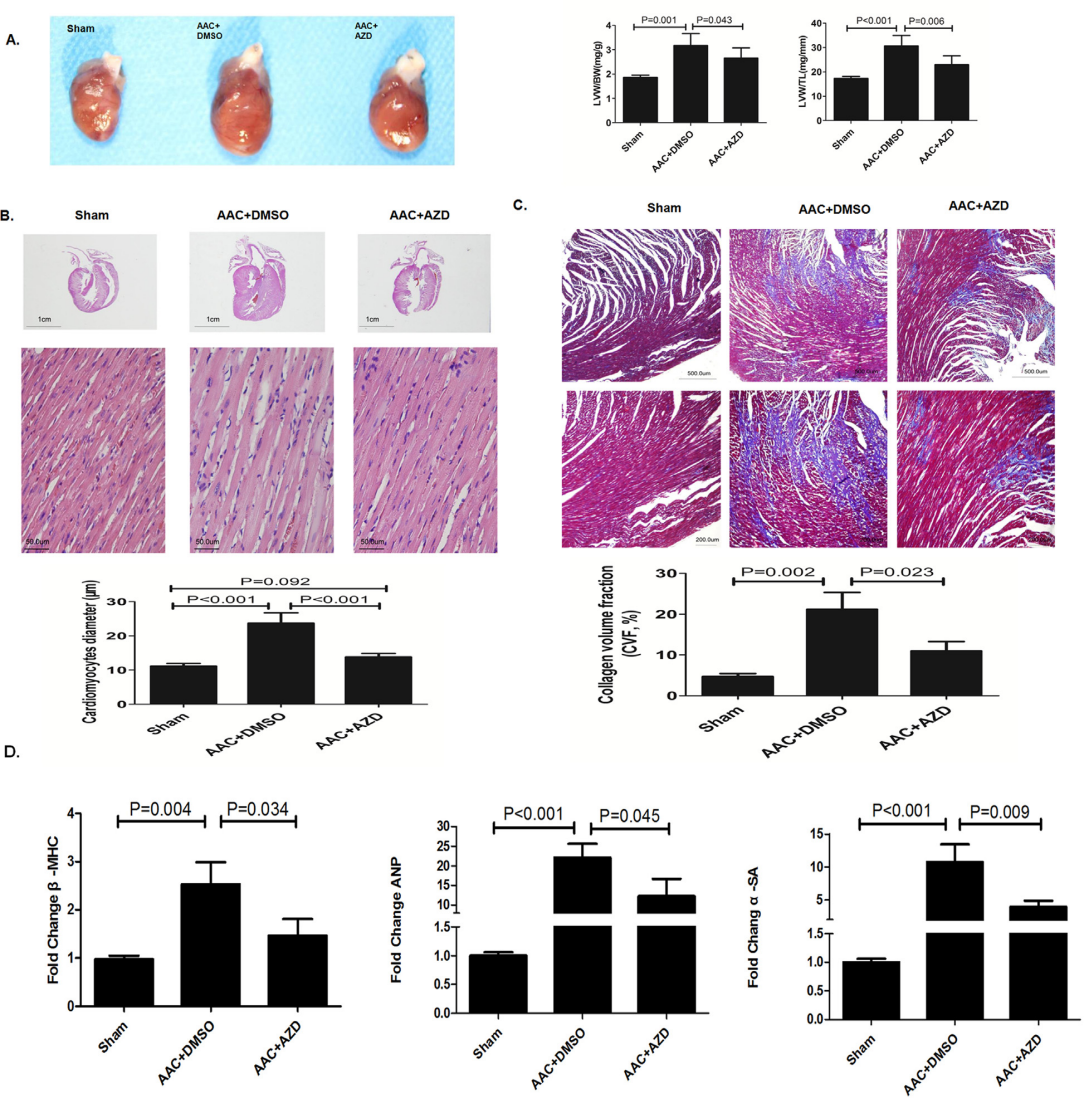
Selumetinib inhibits cardiac hypertrophy in an AAC rat model evidenced by histopathological analysis and gene expression of hypertrophic markers (all figures are from heart sample at 5 weeks’ follow-up). **(A).** Left Panel: representative example of gross heart morphology. Right Panel: the ratio of the left ventricular weight to body weight (LVW/BW) and the ratio of the left ventricular weight to tibia length (LVW/TL). The values are the means ± SD, n = 6. **(B).** Representative histological sections of the LV myocytes stained with hematoxylin and eosin. The bar graph shows the means ± SEM of the cardiomyocyte diameter(μm), n = 6. **(C).** Representative histological sections of LV myocytes stained with Masson’s trichrome. The bar graph shows the means ± SEM of the collagen volume fraction (n = 6). **(D).** The bar graphs indicate the expression levels of β-MHC, ANP, and a-SA measured by real-time RT-PCR in the heart tissue of rats for indicated procedures (n = 5). Values for the real-time RT–PCR were obtained using the ΔΔCT method with GAPDH as a housekeeping gene (similar values were obtained using rp18S as a housekeeping gene). The hypertrophic markers were significantly increased in AAC+DMSO group, and significantly decreased while underwent AZD.

**Fig 8:**
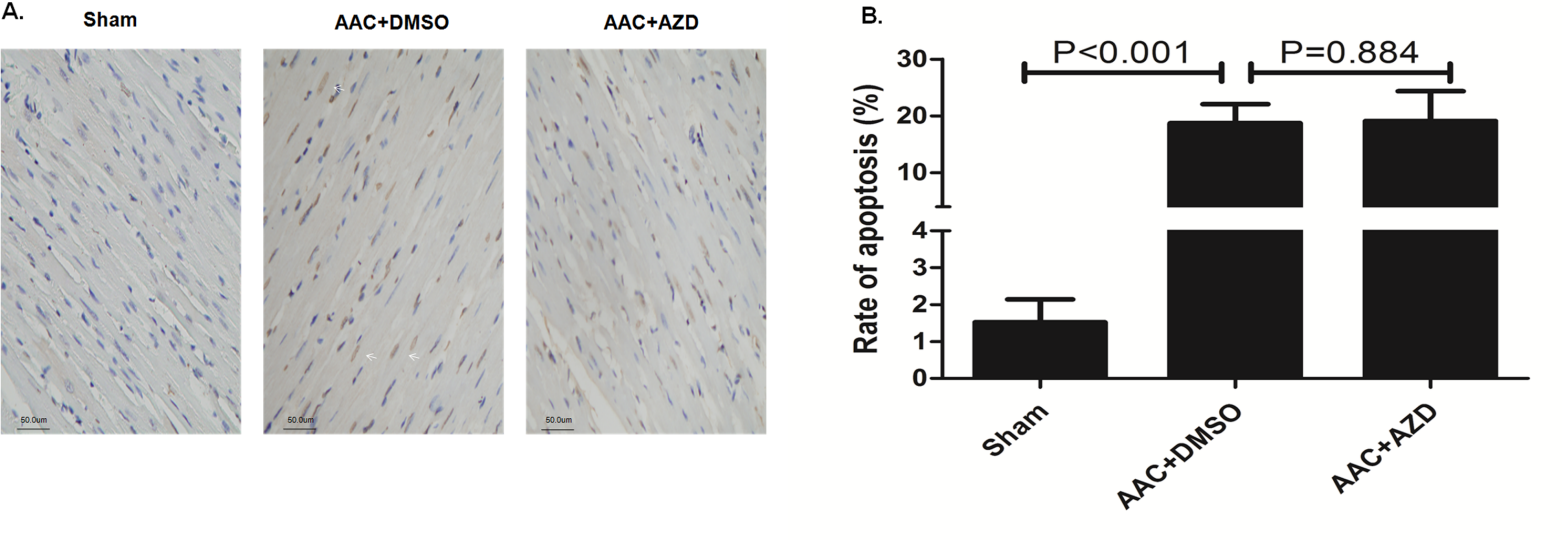
Selumetinib did not increase apoptosis in the AAC cardiac hypertrophic model. **(A).** Representative TUNEL assay in the cardiomyocytes of the AAC rat model at 5 weeks’ follow-up, white arrows indicated the brown apoptotic cells. **(B).** The bar graph shows the means ± SEM of the apoptosis rate, n = 6.

In heart sections harvested after 5 weeks of follow-up, rats that underwent AAC had significantly heavier hearts, while co-treatment with Selumetinib significantly reduced the heart size (Fig 7A). In addition, the cardiomyocytes of rats that underwent AAC were significantly larger than the cardiomyocytes of sham rats or AAC rats receiving Selumetinib (Fig 7B). Moreover, Selumetinib attenuated the over-expression of cardiac hypertrophy markers, including β-MHC, ANP and α-SMA (Fig 7D) as well as prevented cardiac fibrosis caused by AAC pressure overload (Fig 7C). In AAC group, tunnel staining showed higher rate of apoptosis compared with Sham control. However, no incremental apoptosis was observed in AAC rats treated with Selumetinib (Fig 8).

During the longer term follow-up, 2 out of the 4 rats in AAC group died. At the end of 16 weeks follow-up, echocardiography showed rats in AAC group had significantly larger LV dimension and lower left ventricular ejection fraction (LVEF) than control group. Rats treated with Selumetinib had significantly higher LVEF than AAC group (Fig 6D).

### Selumetinib prevents cardiac hypertrophy in swimming rat model

**Fig 9:**
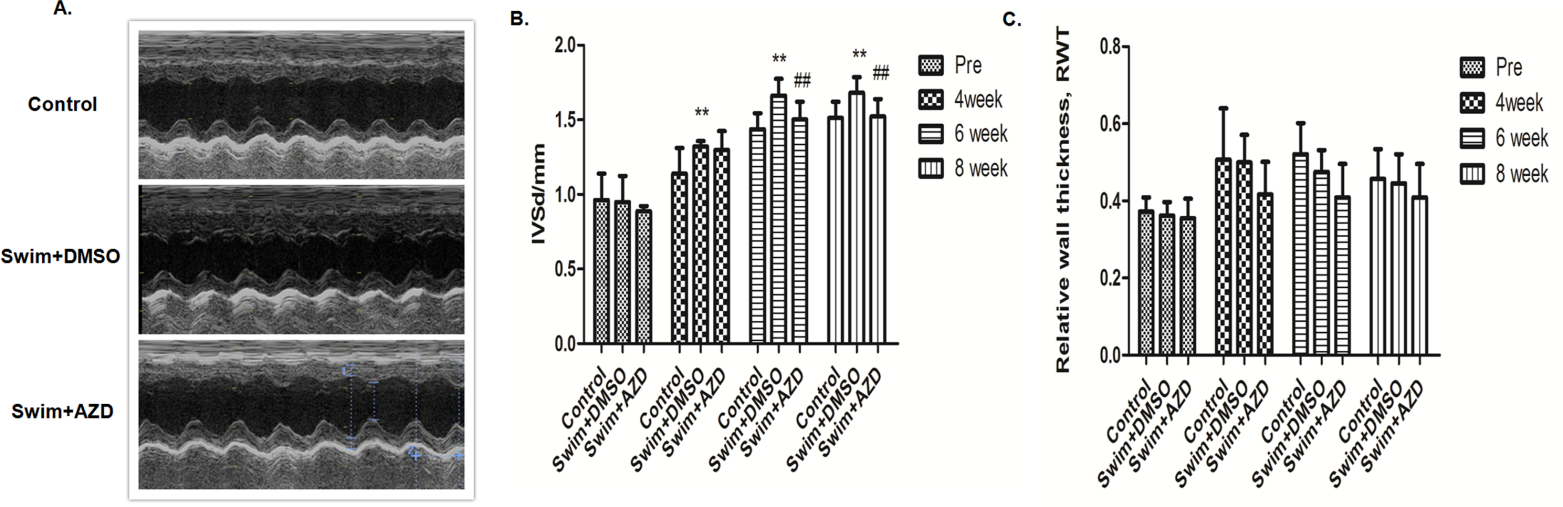
Selumetinib prevents cardiac hypertrophy in a swimming rat model assessed by echocardiographic parameters. **(A).** Representative charts of the trans-thoracic M-mode echocardiogram of the LV at the end of the 8 weeks’ follow-up. **(B).** The diastolic inter-ventricular septal thickness of rats for indicated treatments during follow-up. The values are the means ± SD, n = 10. **: P<0.01 compared with control and ## P<0.01 compared with swim+DMSO. **(C).** The relative wall thickness of rats for indicated treatments during follow-up. The values are the means ± SD, n = 10.

**Fig 10:**
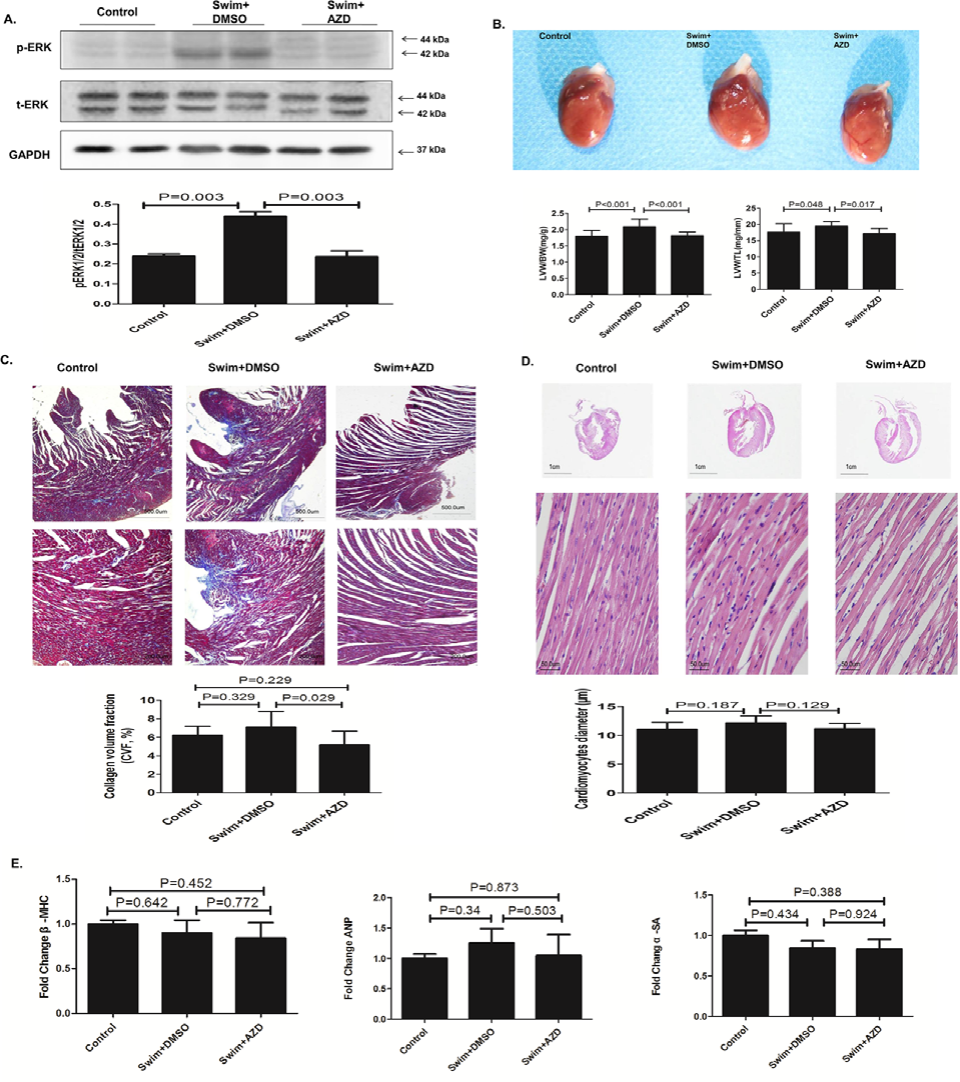
Selumetinib prevents cardiac hypertrophy in a swimming rat model (all figures are from heart sample at the end of 8 weeks’ follow-up). **(A).** Representative immunoblots using antibodies against pERK1/2 and tERK1/2 to probe proteins extracted from the heart of rats for indicated treatments. The bar graph shows the means ± SEM signals pERK/tERK (relative expression from immunoblots of n = 5). **(B).**Upper Panel: representative example of gross heart morphology. Lower Panel: the ratio of the left ventricular weight to body weight (LVW/BW) and the ratio of the left ventricular weight to tibia length (LVW/TL). The values are the means ± SD, n = 10. **(C).** Representative histological sections of LV myocytes stained with Masson’s trichrome. The bar graph shows the means ± SEM of the collagen volume fraction, n = 5. **(D).**Representative histological sections of LV myocytes stained with hematoxylin and eosin. The bar graph shows the means ±SEM of the cardiomyocyte diameter(μm), n = 5. **(E).** Bar graphs indicate the expression levels of β-MHC, ANP and α-SMA in the heart tissue of rats for indicated treatments (n = 5).

**Fig 11:**
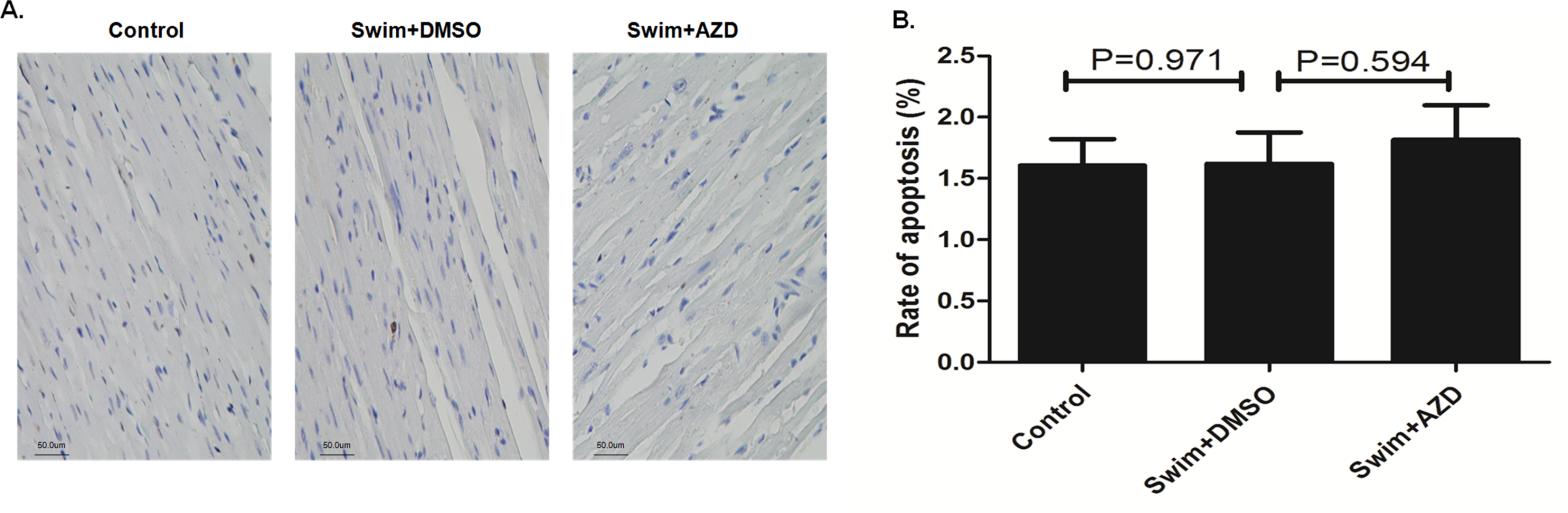
Selumetinib did not increase apoptosis in the swimming rat model. **(A).** Representative TUNEL assay in the cardiomyocytes of a swimming rat model at the end of 8 weeks’ follow-up. **(B).** The bar graph shows the means ± SEM of the apoptosis rate, n = 10.

We also tested the anti-hypertrophy effect of Selumetinib in swimming rats, a widely used animal model for physiologic cardiac hypertrophy. The results were generally similar to those obtained with the AAC pathological hypertrophy model. Swimming rats had significant cardiac hypertrophy phenotypes, including increasing the heart weight, thickening the LV wall and higher ERK activity in the heart (Figs 9 and 10, Table 2). Selumetinib significantly reduced the cardiac ERK activity in the swimming rats (Fig 10A), and, as a result, rescued the cardiac hypertrophy phenotype (Fig 10B). However, unlike the AAC model, there was no significant fibrosis, increasing cardiomyocytes size, apoptosis or fetal gene reactivation (Fig 10C–10E and Fig 11).

**Table 2:** The changes in the echocardiogram parameters for rats in the swim group.

Item	Control	Swim+DMSO	Swim+AZD
HR(bpm)	359 ± 45	364 ± 63	359 ± 51
IVSd(mm)	1.51 ± 0.11	1.68 ± 0.10*	1.50 ± 0.12^#^
LVPWd(mm)	1.46 ± 0.18	1.66 ± 0.11*	1.48 ± 0.19^#^
LVIDd(mm)	6.94 ±0.87	7.00 ± 0. 74	7.40 ± 0.86
LVEDV(ml)	0.78 ± 0.26	0.79 ± 0.24	0.93 ± 0.29
EF%	77.83 ± 9.49	76.54 ± 4.97	75.91 ± 7.93

The changes in the echocardiogram parameters for rats in the swim group at the end of the 8 weeks’ follow-up. The values are given as the mean ± SD. n = 10.

*: p<0.05 compared with control

#: p<0.05 compared with swim+DMSO.

HR: heart rate; IVSd: end-diastolic inter-ventricular septal thickness; LVPWd: end-diastolic left ventricular posterior wall thickness; LVIDd: end-diastolic left ventricular internal dimension; LVEDV: left ventricular end-diastolic volume; and EF: ejection fraction.

## Discussion

The main findings of our study was that Selumetinib, an oral oncology drug targeting the ERK pathway, could attenuate cardiac hypertrophy *in vitro* and *in vivo*, regardless of whether the etiology was chemical stimulus or mechanical overload.

The ERK pathway was, for a long time, considered a potential therapeutic target for cardiac hypertrophy[17]. However, the ERK pathway functions as a critical regulator of cellular differentiation, proliferation, stress responsiveness, and apoptosis[18]. Therefore, global ERK inhibition may result in a variety of off-target side-effects. The safety of long-term administration of conventional ERK inhibitors was of great concern. To the best of our knowledge, there is no currently available ERK inhibitor for clinical use as a cardiovascular drug. The ERK pathway is also involved in oncogenesis and a number of novel ERK inhibitors have been under clinical development for treating cancer[13]. Among those novel drugs, Selumetinib is a very promising oral MEK-1/2 inhibitor. A number of recent Phase II clinical trials have confirmed that Selumetinib is well tolerated in human patients [19–25]. Manageable adverse events, including rash, fatigue, nausea, diarrhea and xerostomia, are common, but no severe adverse effects were noticed. Because of the good safety profile, a phase III clinical trial, named The SELumetinib Evaluation as Combination Therapy-1 (SELECT-1) study, was initiated.

Interestingly, a recent study reported a significant gain in skeletal muscle in 84% of patients receiving Selumetinib[26]. The muscle gain could not be fully explained by the improvement of cancer cachexia because no gain in adipose tissues was observed. Although the underlying mechanism has not been defined, the unexplained muscle gain suggests that Selumetinib might have direct effects on striated muscles. Several recent animal studies have confirmed that Selumetinib could inhibit the aberrantly activated ERK pathway in skeletal and cardiac muscles in a Lmna^*H222P/H222P*^ mice model, improving the muscular phenotype[15,27]. Another study from an independent lab also showed that Selumetinib could attenuate the cardiac phenotype caused by Lmna R225X mutation in patient-specific induced pluripotent stem cell-derived cardiomyocytes[28]. Importantly, unlike other non-selective ERK inhibitors, no severe renal or hepatic side-effects were observed even when a relatively high dose of Selumetinib was used [15]. These pieces of evidence strongly suggest that Selumetinib could serve as a safe and effective cardiovascular drug targeting aberrant ERK activation.

As mentioned before, ERK activation plays a crucial role in not only *LMNA* cardiomyopathy but also in cardiac hypertrophy. As a result, it is of great interest and clinical importance to investigate the efficacy of Selumetinib in cardiac hypertrophy. In our study, we have reported, for the first time, that Selumetinib has promising protective effects against cardiac hypertrophy. Selumetinib significantly inhibit ERK pathway *in vitro* and *in vivo*. Selumetinib treatment prevented cardiomyocyte enlargement, fetal gene overexpression and cardiac fibrosis(Fig 7). Sequential echocardiographic follow-up showed that the anti-hypertrophy and anti-fibrosis effects achieved by Selumetinib were translated into improved concentric remodeling, as evidenced by the significantly lower inter-ventricular septum and relative wall thickness in the AAC rat model(Fig 6). Importantly, an epidemiology study showed that concentric remodeling is a strong prognostic factor in the presence of pressure overload[29]. Longer term follow-up showed the anti-hypertrophy effect of Selumetinib could translate into better LVEF and probably lower mortality(Fig 6D).

It is widely appreciated that cardiac hypertrophy is a heterogeneous pathophysiological condition. To further confirm the anti-hypertrophy effect of Selumetinib, we established another animal model of swimming exercise induced cardiac hypertrophy. The pressure overload-induced pathological hypertrophy is associated with extensive cardiac fibrosis, reactivation of several fetal genes and depressed contractile function. In contrast, physiological hypertrophy after exercise training has a distinct signaling cascade [30]. In our swimming hypertrophy model, aberrant activation of ERK pathway was also noticed. ERK inhibition using Selumetinib was effective in preventing the increasing of LV mass and septal thickness (Figs 9 and 10). The fact that targeting ERK using Selumetinib was effective in preventing cardiac hypertrophy in both the AAC pathological and swimming physiological hypertrophy models strongly indicated that the ERK pathway is a crucial central mediator in the complicated cardiac hypertrophy signaling network.

Side-effects were a major concern of ERK inhibition therapy as this pathway is a very complicated signaling cascade. Consequently, different agonistsor antagonists can produce exquisitely specific cellular biological effects depending on the kinetics of their activation and inactivation, subcellular localization of the kinases, complexes in which they act, and availability of the substrates[31]. Therefore, the cardiac protection effect and side-effects vary depending on the specific ERK inhibition approach used[32,33]. Because of the functional importance and complicated regulation of the ERK pathway, cautiously choosing an appropriate ERK inhibition approach is the key to achieving ideal treatment. For example, a previous study has shown that excessive global ERK inhibition by cardiac-specific expression of a dominant negative form of Raf-1 dramatically increased mortality in the transverse aortic constriction mice model due to the pressure overload-induced apoptosis in cardiomyocytes [34]. This result strongly suggests that ERK activation was initially an adaptive response, which was essential for cardiomyocyte survival under stress. The prolonged and inappropriate ERK activation caused the unfavorable cardiac hypertrophy.

In contrast to the radical gene modification approach, Selumetinib offered relatively mild ERK inhibition. A previous study reported that Selumetinib only has strong ERK inhibition effects on cell lines with elevated ERK activity[14]. Similar results were observed in our study. More importantly, we found that Selumetinib had different effects on normal and stressed cardiomyocytes. In our in vitro cell study, the ERK inhibition effect of Selumetinib was much more extensive in hypertrophic NRCs treated with PE than normal NRCs (Fig 2). This differential ERK inhibition effect of Selumetinib on normal and stressed cells was very important. While extensive ERK inhibition was usually detrimental, Selumetinib theoretically should have minimal adverse effects for normal cells in its therapeutic dose range. This might partially explain its relatively good safety record in clinical trials.

Although the safety of Selumetinib was tested in a number of clinical trials, we should notice that patients with baseline heart diseases were usually excluded from these trials. Unlike the mutation induced ERK activation in cancers, ERK activation was sometimes an adaptive reaction in cardiovascular diseases. Thus, fine-tuning ERK level was crucial. A previous study reported that extensive ERK inhibition could promote apoptosis in cardiomyocytes under stress [34]. Another interesting study reported that genetic deletion of all ERK1/2 from the mouse heart lead to eccentric hypertrophy after angiotensin II/phenylephrine infusion and rapid lethality after transverse aortic constriction [35]. However, in our study, no incremental apoptosis was observed after treatment with Selumetinib in healthy or stressed cardiomyocytes. In our AAC animal models, although a statistically insignificant trend towards left ventricle dilation was noticed in the early stage (Table 1.), long-term survival and LVEF was improved after Selumetinib treatment (Fig 6D). Our *in vitro* and *in vivo* results strongly suggested that the differential ERK inhibition effect is very important for the safety of long-term Selumetinib administration.

In addition to the ERK inhibition level, off-target effect was another important safety concern. Previous studies have already demonstrated that Selumetinib was a very selective MEK 1/2 inhibitor. Selumetinib specifically inhibit ERK 1/2 but has a minimal effect against other kinases, including other MAPK family members[14]. In our study, The conventional ERK inhibitor PD98059 target not only ERK pathway but also AKT and calcineurin. In contrast, Selumetinib had a very selective ERK inhibition effect in stressed cardiomyocytes(Fig 4). This high selectivity was also important for its clinical safety.

In conclusion, our preclinical results showed that Selumetinib, a novel oral anti-cancer drug with good safety records from a number of phase II clinical trials, can inhibit ERK activity in the heart and prevent cardiac hypertrophy in response to stress. These promising results implicate that Selumetinib could potentially be used to treat cardiac hypertrophy. However, this hypothesis needs to be validated in human clinical trials.

## Study Limitation

The systolic heart failure in AAC rat model was not apparent until 16 weeks' follow-up. Our study was mainly designed to test the anti-hypertrophy effect of selumetinib. Only a relatively small number of animals were included in the longer-term follow-up. And 2 out of 4 rats in the AAC group died at the end of 16 weeks follow-up. This could induce selection bias in the statistical analysis. Future studies were needed to further investigate whether Selumetinib could prevent heart failure caused by pressure overload and other common causes.

## Supporting Information

S1 Supplemental methods (DOCX)
