# Supplementary material for: Selumetinib, an Oral Anti-Neoplastic Drug, May Attenuate Cardiac Hypertrophy via Targeting the ERK Pathway
Source: PLoS One. 2016 Jul 20;11(7):e0159079. doi: 10.1371/journal.pone.0159079 (PMC4954659; doi:10.1371/journal.pone.0159079)
Supplement: S1 Supplemental methods — (DOCX) [file pone.0159079.s001.docx]

**Supplemental Material**

***Selumetinib, an oral anti-neoplastic drug, may prevent cardiac hypertrophy via targeting the ERK pathway***

**Chen Li****^1^**^¶^**, Zhongxiu Chen****^1^**^¶^**, Hao Yang^1^, Fangbo Luo****^2^,** **Lihong Chen^3^,** **Huawei Cai^4^, Yajiao Li^1^, Guiying You^1^, Dan Long****^5^, Shengfu Li^5^, Qiuping Zhang^6^, Li Rao^1＊^**

**^1^** Department of Cardiology, West China Hospital of Sichuan University, Chengdu, Sichuan, China; ^2^ Department of Rehabilitation, Community Health Center of Shuangnan Wuhou District, Chengdu, Sichuan, China; ^3^ Department of Biochemistry and Molecular Biology, West China School of Preclinical and Forensic Medicine, Sichuan University, Chengdu, Sichuan, China; ^4^ Department of Nuclear Medicine, West China Hospital of Sichuan University, Chengdu, Sichuan, China; ^5^ Key Laboratory of Transplant Engineering and Immunology, West China Hospital of Sichuan University, High-tech Zone, Chengdu, Sichuan, China; and ^6^ King's College London British Heart Foundation Centre of Research Excellence, Cardiovascular Division, London, UK.

^¶^:These authors contributed equally to this study.

***:** Corresponding author E-mail: [lrlz1989@163.com](mailto:lrlz1989@163.com)（LR）

**Supplemental methods**

**Cell culture**

The neonatal rats were sacrificed using cervical dislocation method. The hearts of 1- to 2-day-old neonatal Sprague-Dawley (SD) rats were cut into 1 mm^3^ and digested with 0.05% (w/v) collagenase II (GIBCO, USA) and 0.05% (w/v) trypsin (TN, GIBCO, USA) to digestive turbidity, repeating until the tissue blocks disappeared. The digests were resuspended in H-DMEM (GIBCO, USA) supplemented with antibiotics (GIBCO, USA) and 10% fetal bovine serum (GIBCO, USA). Fibroblasts were removed by pre-incubation for 30 min at 37℃. The cardiomyocytes in suspension were collected and seeded in a 6-well plate containing H-DMEM supplemented with 10% FBS, 100 mM BrdU (Sigma) for 24 h before subsequent experiments. The purity of the cardiomyocytes assessed by immunofluorescence analysis with an antibody against sarcomeric a-actinin (Santa Cruz, USA) was above 90%.

**Cell area measurement**

After washing with phosphate-buffered saline (PBS), adherent cells were fixed with 4% paraformaldehyde (Analytical Reagent) in PBS for 30 min and stained with 0.1% Crystal violet (Sigma) for 10 min. Images were obtained using a digital camera attached to an inverted microscope (OLYMPUS IX71, Japan) for analysis. Four random photographs were collected from each sample, and at least 90 individual cells were examined in each group. The cell size was analyzed using Image-Pro PLUS software (MediaCybernetics, Silver Spring, MD, USA). The data shown represent the image analysis from three independent experiments.

**AAC and swimming hypertrophy models**

All of the protocols and surgical procedures were in accordance with the Guide for the Care and Use of Laboratory Animals published by the US National Institutes of Health (8th edition, 2011). The animal procedures were approved by the Institutional Animal Care and Use Committee of Sichuan University. The animals were housed with 3 to 5 per cage at a controlled roomtemperature (22°C) with a 12-hour dark-light cycle and fed standard rat chow; they also had access to water ad libitum.

The exercise-induced cardiac hypertrophic model was established by swimming. Male SD rats (180 to 220 g, n=30) were randomly divided into the following 3 experimental groups, each with 10 rats:1) Sedentary(control; n=10); 2) Simple swimming(swim+DMSO; n=10); and 3) Swimming treatment group(swim+AZD; n=10). Training consisted of swimming sessions with a 60-minute duration, 6 days a week, for 8 weeks, which were carried out between 11:30 AM and 1:30 PM. The exercise duration was gradually increased until the rats could swim for 60 minutes in the first week. The swimming apparatus measures 120 cm in length, 70 cm in width, 70 cm in height and a water depth of 50 cm. The water temperature were kept between 30-35℃.

Pressure overload-induced cardiac hypertrophy was induced with ascending aortic constriction (AAC) surgery. Male SD (180-200g) rats were anesthetized with 10% chloral hydrate (in 300mg/kg). After the rats were confirmed to be in an anaesthetized state (e.g., no response to toe pinching), they were ventilated by tracheal intubation using a DHX-300 rodent ventilator (Science and Technology Co., Ltd. Thai Union, Chengdu, China) with a tidal volume of 15 mL and a respiratory rate of 100 breaths/min. The chest was opened at the second intercostal space on the left, and the thymus glands were superiorly reflected. The ascending aorta was dissected, and a 6-0 silk suture was tied around the aorta against a 20-gauge needle (o.d. 0.9 mm). Both control groups underwent a sham operation involving thoracotomy and aortic dissection without constriction of the aorta. There were 3 experimental groups (Sham, AAC+DMSO and AAC+AZD, each with 10 rats) of the AAC hypertrophy rat model.

**Transthoracic echocardiography**

Cardiac hypertrophy and function were assessed by echocardiography before and after AAC surgery or a swimming exercise-induced model using a Vivid 7 Dimension (GE Medical Systems, USA) equipped with a 10S phased array probe (Frequency 4.0-11.0 MHz) under anesthesia with 1.5% isoflurane, allowing for spontaneous breathing. The relative wall thickness (RWT) was calculated by 2 LVPWd divided by LVIDd.

**Measurement of Cardiac Hypertrophy**

The rats were anesthetized with 10% chloral hydrate (in 300mg/kg by ip). After the rats were confirmed to be in an anaesthetized state (e.g., no response to toe pinching), the jugular vein was dissected upside the collarbone, and the hearts were stopped at diastole by the injection of 10% KCl in the jugular vein for measurement the cardiac hypertrophy phenotype. After the heart was weighed, the left ventricle (LV) was dissected, corresponding to the remaining tissue upon removal of both the atria and free wall of the right ventricle. The interventricular septum remained as part of the LV. Cardiac hypertrophy was assessed by measuring the ratio of the LV weight in milligrams to animal body weight (BW) in grams (LVW/BW in mg.g^-1^ ) and to the tibia length (TL) in millimeters (LVW/ TL in mg.mm^-1^).

**Quantitative real-time RT–PCR analysis**

Total RNA was extracted from cardiomyocytes and ventricular tissue using RNAiso plus (TaKaRa, Japan) according to the manufacturer’s instruction. The RNA concentration was quantiﬁed with a NanoDrop 2000 spectrophotometer(Thermo Scientific, USA). cDNA was synthesized from total RNA using a PrimeScript^TM^ RT reagent Kit with gDNA Eraser (TaKaRa Bio Inc, JAPAN) in S1000TM Thermal Cycler (BIO-RAD, USA). The amount of cDNA corresponding to 100 ng of RNA was ampliﬁed using SYBR Premix Ex Taq^TM^ II (TaKaRa Bio Inc, JAPA), with the primers for rat beta-myosin heavy chain (β-MHC), atrial natriuretic peptide(ANP), skeletal alpha-actin (α-SA), glyceraldehyde phosphate dehydrogenase (GAPDH), and ribosomal protein 18s. The primers (Table S1) were purchased through Invitrogen. Real-time PCRs were performed, recorded, and analyzed with the C1000TM Thermal Cycler (CFX96TM Rea-Time System, Bio-Rad, USA).

Table S1. Primer sequences used for real-time quantitative RT-PCR analysis.

| β-MHC | 5′-TTGGCACGGACTGCGTCATC-3′(forward) |
| --- | --- |
|  | 5′-GAGCCTCCAGAGTTTGCTGAAGGA-3′ (reverse) |
| ANP | 5′-ATCTGATGGATTTCAAGAACC-3′(forward) |
|  | 5′-CTCTGAGACGGGTTGACTTC-3′(reverse) |
| a-SA | 5′-TCGCGACCTTACTGACTACCTG-3′ (forward) |
|  | 5′-GCTTCTCTTTGATGTCGCGC-3′(reverse) |
| GAPDH | 5′- GAGAAGGCTGGGGCTCAC-3′(forward) |
|  | 5′- GTTGTCATGGATGACCTTGGC-3′(reverse) |
| rp18S | 5′- CCTTCGCTATCACTGCCATT-3′(forward) |
|  | 5′- TGGCCAGAACCTGGCTATAC-3′(reverse) |

**Western blot analysis**

Cells were washed with cold PBS, centrifuged, and lysed on ice for 30 minutes in RIPA lysis buffer containing protease and phosphatase inhibitors. The protein concentrations were determined with the BCATM protein assay kit (Thermo Scientific pierce, USA). Cellular protein extracts (30μg) were loaded in 12% SDS-polyacrylamide gel electrophoresis, and transferred electrophoretically onto PVDF membranes (0.45 μm). Nonspeciﬁc sites were blocked in 5% (w/v) nonfat milk powder in PBST buffer [0.01 M PBS, pH 7.2-7.4, containing 0.05% (v/v) Tween-20] for 1 h at room temperature. Then, the membranes were incubated with the appropriate primary antibody(1:1000) overnight at 4°C. After three 10-minute washes in PBST, the membranes were incubated with HRP-conjugated secondary antibody (1:4000) in the blocking buffer for 60 min at 37°C. After three 10-minute washes in PBST, the proteins were detected by ImmobilonTM Western Chemiluminescent HRP substrate (Millipore, USA). pERK1/2, ERK1/2 and GAPDH antibodies were purchased from Cell Signaling Technology. pAKT, tAKT were purchased from Zen Bioscience (Chengdu, China), and the phosphorylation site is 308. For quantiﬁcation of the phosphorylated ERK1/2 compared with the total ERK1/2, immunoblots were analyzed using Quantity one (Bio-Rid, USA). Heart, liver, lung or kidney tissue from the rats was homogenized in extraction buffer, and the method of Western blot analysis was similar to that for cells.

**Histopathological analysis**

Hearts from the AAC and swimming rats were ﬁxed in 4% paraformaldehyde for 48 h, embedded in parafﬁn, sectioned at 5 µM, and stained with hematoxylin and eosin and Masson’s trichrome. A terminal deoxynucleotidyl transferase dUTP nick end labeling (TUNEL) assay was performed using a FragEL™ DNA Fragmentation Detection Kit, Colorimetric -TdT Enzyme (Merck Calbiochem, Germany) according to the manufacturer’s protocol. Representative stained sections were photographed using a light microscope (OLYMPUS BX51, Japan). Images were processed using Adobe Photoshop CS (Adobe Systems). For quantiﬁcation of the cardiomyocyte diameter, myocardial ﬁbrosis, and cell apoptosis, micrographs of sections stained with hematoxylin and eosin, Masson’s trichrome, and TUNEL were measured (Image-Pro PLUS software).

# The ERK inhibition effect with AZD6244 was significant in neoplastic cells and mild in non-neoplastic cells.

MCF7, Colo205, 786-O and Panc02 cells were cultured in RPMI-1640 (GIBCO, USA) supplemented with 10% fetal bovine serum. H9c2 embryonic rat heart-derived cells, human umbilical vein endothelial cells (HUVEC), and BRL-3A rat liver cells were cultured in H-DMEM(GIBCO, USA) supplemented with 10% fetal bovine serum (GIBCO, USA). Cells were grown in an atmosphere of 95% O_2_ and 5% CO_2_ in a humidiﬁed incubator. Stock cultures were passaged at 2- to 3-day intervals. Cells were seeded in six-well plates and treated with AZD6244 for Western blot.
